# Deciphering glycosylation-driven prognostic insights and therapeutic prospects in glioblastoma through a comprehensive regulatory model

**DOI:** 10.3389/fonc.2024.1288820

**Published:** 2024-05-22

**Authors:** Xingyi Jin, Zhuo Chen, Hang Zhao

**Affiliations:** Neurosurgery Department, China-Japan Union Hospital of Jilin University, Changchun, Jilin, China

**Keywords:** glycosyltransferases, glyco-model, GB, immune checkpoint inhibitors, clofarabine

## Abstract

The oncogenesis and development of glioblastoma multiforme have been linked to glycosylation modifications, which are common post-translational protein modifications. Abnormal glycosyltransferase development leads to irregular glycosylation patterns, which hold clinical significance for GB prognosis. By utilizing both single-cell and bulk data, we developed a scoring system to assess glycosylation levels in GB. Moreover, a glycosylation-based signature was created to predict GB outcomes and therapy responsiveness. The study led to the development of an glyco-model incorporating nine key genes. This risk assessment tool effectively stratified GB patients into two distinct groups. Extensive validation through ROC analysis, RMST, and Kaplan-Meier (KM) survival analysis emphasized the model’s robust predictive capabilities. Additionally, a nomogram was constructed to predict survival rates at specific time intervals. The research revealed substantial disparities in immune cell infiltration between low-risk and high-risk groups, characterized by differences in immune cell abundance and elevated immune scores. Notably, the glyco-model predicted diverse responses to immune checkpoint inhibitors and drug therapies, with high-risk groups exhibiting a preference for immune checkpoint inhibitors and demonstrated superior responses to drug treatments. Furthermore, the study identified two potential drug targets and utilized Connectivity Map analysis to pinpoint promising therapeutic agents. Clofarabine and YM155 were identified as potent candidates for the treatment of high-risk GB. Our well-crafted glyco-model effectively discriminates patients by calculating the risk score, accurately predicting GB outcomes, and significantly enhancing prognostic assessment while identifying novel immunotherapeutic and chemotherapeutic strategies for GB treatment.

## 1 Introduction

Glioblastoma (GB) has evolved into a highly threatening and deathly brain tumor, with an overall survival time of 15-17 months as the median (1). GB is characterized by its rapid proliferation and extensive vascularization, which is supported by the tumor’s aggressive growth dynamics promoting angiogenesis (2). Immunotherapy is a potential novel medication that increases anticancer immune responses by controlling T cells’ stimulation and function activities (3). Monoclonal antibodies targeting PD-1/PD-L1 or CTLA-4 have been used in many clinical trials to induce long-lasting therapeutic responses in some cancer patients (4). Therefore, it is of the utmost importance to investigate new markers predicting immunotherapy response and to build solid prognostic signatures for GB patients, enabling the classification of patients and targeted therapy.

Glycosylation is a protein modification progress regulated by glycosyltransferases (5). Several glycosylation modifications, such as O-glycosylation, N-glycosylation, sialylation and fucosylation, are significantly correlated to cancer; these alterations drive multiple cancerous behavior patterns of tumors, including tumor depersonalization, metastasis, and immune regulation (6). A poor prognosis is predicted for glioma patients with the ST3GAL1-associated O-linked sialylation, which also enriches increasing cancer stages in the heterogeneous molecular classification (7). The deregulation of FUT8 contributes to GB tumorigenesis and provides unique insights into the role of fucosylation in receptor tyrosine kinase activity and TMZ resistance (8). Therefore, glycosylation is implicated in numerous aspects of GB oncogenesis, progression, and immune regulation. Emerging data have established those dynamic glycosylation alterations are intimately connected with the course of tumors due to the development of glycomics. Some literature suggests that protein glycosylation is a viable event for diagnosing and tracking a variety of malignancies (9). For example, low MGAT1 expression was associated with liver cancer cell dedifferentiation, metastases, and poor outcomes (10). In addition, Liu et al. found that high levels of GALNT6 expression were correlated with decreased survival rates and that GALNT6 promotes breast cancer metastasis through α2M O-glycosylation (11). Identifying underlying glycosylation biomarkers and expression abnormalities is essential to predict diagnostics, outcomes, and treatment responses for malignancies. For this reason, exploring the role of glycosylation regulators in creating a GB risk prediction model is fascinating.

In the current investigation, we constructed the glyco-score and assessed the GB samples on bulk and single-cell levels. Then we established a glyco-model that predicts GB prognosis, immunotherapy and chemotherapy responses. Survival time, glycosyltransferase expression, tumor microenvironment, immunotherapy response, and chemosensitivity significantly correlate with risk score. Meanwhile, clinical experiments demonstrated that the chosen glycosylation regulators are related to the immunological status and malignant characteristics of GB. Our comprehensive analysis of glycosylation patterns offers promising avenues for GB diagnosis and therapy choice, facilitating a more tailored treatment strategy. By identifying specific glycosylation signatures, we aim to predict patient outcomes and therapy responses more accurately, which is critical in the context of personalized medicine for GB.

## 2 Materials and methods

### 2.1 Data acquisition

We meticulously collected gene profiles, mutational landscapes, and clinical data from the TCGA database, which served as our training set. To ensure a robust dataset, we excluded any samples lacking complete survival information. For validation purposes, additional datasets were retrieved from the CGGA and GlioVis databases, rigorously adhering to similar criteria for data completeness and reliability (12, 13).

### 2.2 Single-cell data processing and analysis

Single-cell data of GB was downloaded from the GEO database under the accession number GSE162631. We removed the genes that were not expressed in every case (counts = 0), then normalized the gene expression matrix using the “SCTransform” function in the Seurat R package. Moreover, we performed the PCA and UMAP analysis and classified the cells using the FindNeighbors and FindClusters functions. Doublets were filtered using the DoubletFinder R package. Cells with > 15% mitochondrial genes or gene number< 500 were also removed. After quality control, about 100 thousand cells were subjected to cell-type annotation using the Celltypist package in Python.

### 2.3 Functional enrichments

The GO and KEGG databases were employed to conduct fully functioning activity and pathway analysis involving the differential expression glycosylation regulators between glioma tumors and normal tissues using the Enrichplot package in R (14, 15). Moreover, using the clusterProfiler algorithm (16), GSEA was used to evaluate the functions between the two risk subgroups. Statistical significance was considered to exist when the FDR< 0.05 after 1,000 permutations.

### 2.4 Establishment of glyco-score

A total of 223 glycosylation regulators were retrieved from the GlycoGene DataBase (GGDB). To explore the glycosylation affections on GB, we performed the differential analysis between the GB and normal tissues in the GTEx-TCGA dataset. The differentially expressed genes were shown in the heatmap, and the gene correlation was analyzed using the igraph package. The glyco-score was assessed based on the differentially expressed glycosylation regulators using the ssGSEA and Ucell algorithms in bulk and single-cell data, respectively (17, 18).

### 2.5 Development and validation of glyco-signature

To determine predictive glycosylation regulators, a univariate Cox regression analysis was conducted on differentially expressed glycosylation regulators in a training set to choose nine glycosylation regulators associated with the GB outcomes. For Cox regression analysis for GB prognosis, the OS of GB patients was examined and computed. Additionally, the lasso regression was leveraged to extract glycosylation regulators and construct a glyco-model for gauging the outcome of GB patients. The mathematical methodology was utilized to ascertain the risk rating:


$$\mathrm{riskscore}=\sum_{i=1}^{n}\left(\beta_{i}\times Exp_{i}\right)$$

Where n is the glycosylation regulator counts; Exp is the glycosylation gene profile; β indicates the multi-Cox coefficient. Patients were then classified into different risk subgroups according to their risk scores. Moreover, the external sets were used to examine the generality of the risk characteristic. Using R v4.2 and Kaplan–Meier (KM) survival analysis, the variation in outcome between the two risk subgroups was determined to be statistically significant (P< 0.05).

### 2.6 Assessing risk model reliability and generating nomogram

Prognosis analysis assessed the difference between glyco-model and common characteristics, including age, gender, and grades. In the forest plots, P-values and HR were displayed. A nomogram was established using a glyco-model and selected characters in the rms R package to assess three-time points’ OS in GB patients. To assess the reliability of our glyco-model, we integrated it with demographic and clinical factors using multivariable Cox regression analyses to develop a comprehensive nomogram. This tool projects 1-, 3-, and 5-year survival probabilities for GB patients, employing calibration plots and AUC curves to evaluate predictive accuracy.

### 2.7 Analysis of immune infiltration

The ssGSEA algorithm was employed to calculate 20 critical pathways using the gsva R package (19), and CIBERSORT was leveraged to specify the cells in the tumor microenvironment (TME) (20). We further quantified the stromal score, immunological score, and tumor purity using the ESTIMATE algorithm (21).

### 2.8 Estimation of drug target

We acquired comprehensive target data for 6,125 compounds from the Drug Repurposing Hub (https://clue.io/repurposing) and got 2,249 unique drug targets following the elimination of duplicates (22). To isolate genes amenable to targeting, holding potential for therapeutic implications in high-risk GB patients, we initially conducted Spearman correlation analysis. This assessment involved correlating gene expression of targetable genes with risk scores. Any gene exhibiting a correlation coefficient exceeding 0.25 (with a significance level of P< 0.05) was identified as a candidate drug target associated with an unfavorable prognosis. Subsequently, we determined the risk score for brain cell lines from the CCLE project. We then undertook a correlation analysis between the CERES score and risk score, utilizing these specific cell lines. CERES represents a method used to estimate gene dependency while compensating for the impact of copy-number variations. The Avana dataset applies this methodology to calculate CERES scores for every gene and cell line (23). A lower CERES score for a particular gene suggests an increased likelihood of its dependency on a given cancer cell line. Hence, genes displaying a correlation coefficient below -0.2 (with P< 0.05) were categorized as drug targets linked to poor prognosis dependence. Consequently, therapeutic drug targets suitable for high-risk score GBs encompassed those identified through both aforementioned analyses.

### 2.9 Chemotherapeutic response prediction

Two extensive pharmacogenomic datasets, namely CTRP and PRISM, offer expansive drug screening and molecular data spanning numerous cancer cell lines. This extensive dataset facilitates precise prognostication of drug response in clinical samples. Distinct differential expression analyses were conducted both between bulk samples and cell lines and between the samples and cell lines, respectively.

For the task of predicting drug responses, a plethora of machine learning (ML) methods have been documented, encompassing multivariate linear regression, support vector machine (SVM), random forests (RF), and k-nearest neighbors (KNN). Among the array of ML methods, linear regression techniques, such as ridge regression and elastic net, have demonstrated consistent and robust performance across diverse contexts (24). Therefore, the present study employed the ridge regression model encapsulated within the pRRophetic package. This model, which has demonstrated reliability across multiple studies, was employed to forecast drug responses for clinical samples (25, 26). Training of the predictive model relied on expression profiles and drug response data derived from solid Cancer Cell Lines (CCLs), with the exclusion of hematopoietic and lymphoid tissue-derived CCLs. The predictive model exhibited satisfactory performance assessed through default 10-fold cross-validation, facilitating the estimation of drug responses for clinical samples based on refined expression profiles.

### 2.10 Connectivity map analysis

As a supplementary approach, the analysis of the Connectivity Map (CMap) was conducted to explore the potential therapeutic utility of candidate agents in GB (27). Initially, a comparative analysis of gene expression was executed between samples from tumor and normal tissue. Subsequently, the top 300 genes exhibiting the most pronounced fold changes (including 150 up-regulated genes and 150 down-regulated genes) were submitted to the dedicated CMap website, accessible at https://clue.io/query. The gene expression signatures utilized by this platform are sourced from a combination of CMap v1 and the Library of Integrated Network-Based Cellular Signatures (LINCS) database. Remarkably, the CMap analysis incorporates a comprehensive selection of 2,429 compounds. The outcome of this analysis generated a distinct connectivity score for each perturbation, calibrated on a standardized scale that ranges from -100 to 100. Significantly, a negative score indicates a gene expression pattern linked to a specific perturbation that runs contrary to the disease-specific expression pattern. This implies the potential therapeutic efficacy of the respective perturbation within the context of the disease.

### 2.11 Clinical sample collection and patient stratification

This study employed human specimens obtained from a cohort of 20 patients diagnosed with GB. These specimens were procured from patients undergoing surgical procedures at China-Japan Union Hospital. All collected materials were subjected to HE staining, following established protocols. Notably, two distinct pathologists independently conducted the diagnostic assessments.

Total RNA was extracted using the Trizol method (Invitrogen), which is widely recognized for its efficiency. The quantitative real-time PCR (qRT-PCR) was conducted using the One-Step qPCR Kit (Invitrogen) and the CFX Connect™ Real-Time System (BIO-RAD), strictly following the protocols provided by the manufacturers. For data analysis, we used the 2^-ΔΔCq^ method, normalizing gene expression levels to GAPDH as a reference. This normalization is critical for ensuring consistency across samples. Based on these gene expression levels, patients were stratified into low-risk and high-risk groups using a threshold calculated from the glyco-model’s equation, which helps in predicting patient outcomes more accurately.

### 2.12 Histological evaluation

To prepare glioma tissue sections for immunohistochemistry (IHC), we deparaffinized and rehydrated the sections in a series of gradient ethanol and recovered them by heating the slides at 100°C citrate buffer for 1 hour. Then, the slices were incubated with the primary antibodies and HRP-conjugated secondary antibodies in sequence. DAB Peroxidase Substrate Kit was used to visualize the antigen-antibody combination. The IHC images were acquired utilizing a microscope. Immunohistochemistry was performed using antibodies against CD3 (ab16669, Abcam), CD57 (ab82749, Abcam), CD163 (ab79056, Abcam) and FOXP3 (ab20034, Abcam).

## 3 Results

### 3.1 Potential role of glycosylation regulators in GB

**Figure 1:**
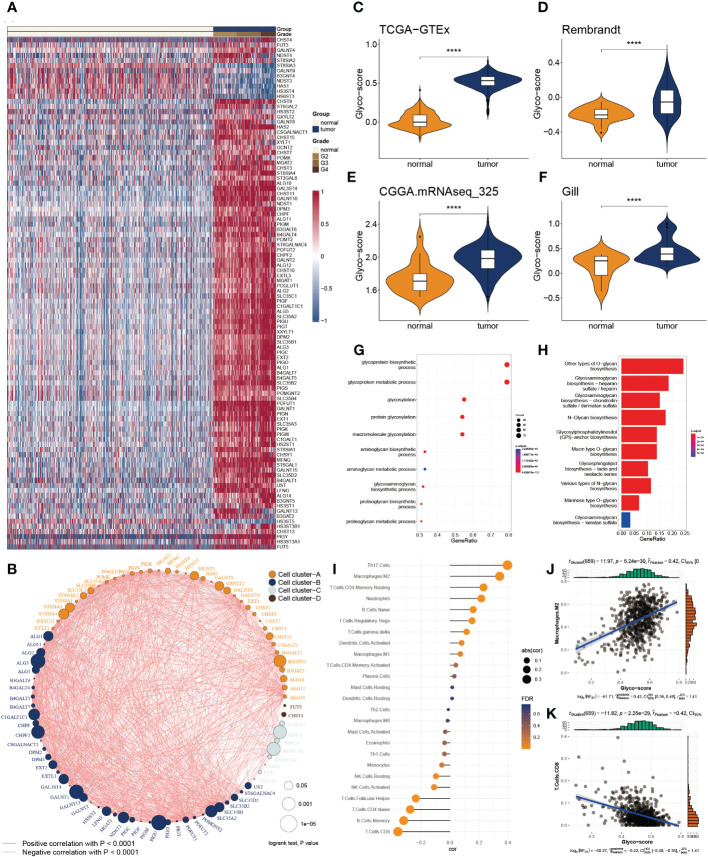
Functional enrichment and prognostic characters of glycosylation regulators. **(A)** Heatmap showing the 100 differential glycosylation genes in glioma. **(B)** Interactive correlation of top 50 glycosylation genes. **(C-F)** Distribution of glyco-score in four datasets, GTEx-TCGA **(C)**, Rembrandt **(D)**, CGGA.mRNAseq_325 **(D)** and Gill **(F)**. **(G)** GO enrichment of differential glycosylation regulators. **(H)** KEGG analysis of differential glycosylation regulators. **(I)** Correlation of glyco-score with immune cell inflictions. **(J)** Representative positive correlation: M2 macrophage. **(K)** Representative negative correlation: CD8^+^ T cells. ****P < 0.0001.

Of 223 glycosylation regulators, 100 genes were abnormally expressed in GB patients compared with the normal tissues, indicating a significant variation in biological processes between the GB patients and healthy individuals. Heatmap demonstrated the landscape of 100 differential genes between the two groups. Therein, we observed that 8 genes were downregulated in GMB patients, while 92 genes were dramatically increased in GB relative to the normal cases (**Figure 1A**). To systemically explore the relationship among the 100 differential genes, we classified them into four clusters and constructed the correlation network. We detected a strong association of 100 genes, for instance, in cluster A, EXT2 and POFUT1 are highly synergistic (r = 0.798), whereas GAL3ST4 and ST8SIA3 from cluster B are antagonistic (r = -0.562). Moreover, we observed the highest association between ALG11 and POMK (r = 0.802) and the remarkably converse correlation between GALNT13 and HS3ST3B1 (r = -0.581) (**Figure 1B**).

To delineate the communication of glycosylation with GB, we estimated the glyco-score for every GB patient using the ssGSEA algorithm established from the differential glycosylation regulators. We found that the glyco-score was dramatically higher in GB than in normal cases, validated in the other three datasets (Rembrandt, Gil, and CGGA.mRNAseq_325) (**Figures 1C-F**). Furthermore, functional investigations were employed to determine the physiological activities of glycosylation regulator-associated differentially expressed genes. In **Figure 1G**, a distribution chart illustrates the top 10 enriched GO terms of the molecular mechanism for glycosylation regulators. These concepts were linked with glycosylation, glycoprotein biosynthesis, and glycoprotein metabolism. As shown in **Figure 1H**, KEGG analysis revealed that glycosylation regulators were abundant in O-glycan biosynthesis, glycosaminoglycan biosynthesis, and N-Glycan biosynthesis. These results implied that the mutual effect of the differential glycosylation regulators might be the significant reason for triggering GB.

Since TME is involved in tumor formation, we evaluated the relationship between the glyco-score and immune infiltration (**Figure 1I**). We found a strong positive correlation of glyco-score with M2 macrophages but a negative association with CD 8^+^ T cells (**Figures 1J, K**), indicating the antagonistic effects of glyco-score in shaping the hot TME of GB.

### 3.2 Evaluation of glyco-score at the single-cell level

**Figure 2:**
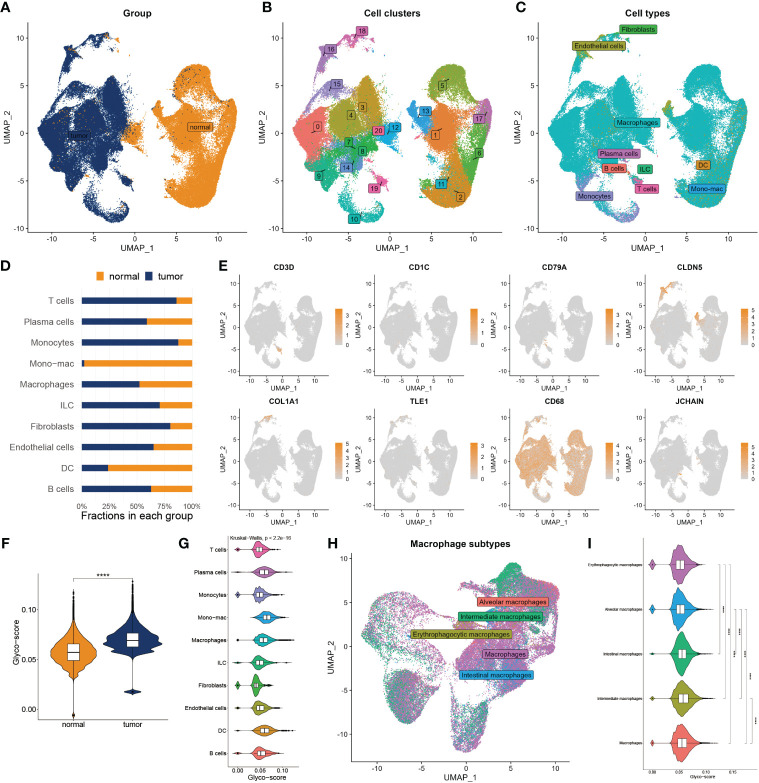
Landscape of glyco-score in single-cell sequence. **(A)** Cell distribution in tumor vs normal tissues. **(B)** Distribution of cell cluster. **(C)** Distribution of cell annotation. **(D)** Cell proportions in tumor vs. normal tissue. **(E)** Representative immune marker in each cell type. **(F)** Distribution of glyco-score. **(G)** Glyco-score correlates immune cell types. **(H)** Distribution of cell annotation in macrophage subtypes. **(I)** Glyco-score correlates with macrophage subtypes. ****P < 0.0001.

To deeply explore the TME variation between GB and normal individuals, we analyzed single-cell data from GB patients in-depth. After quality control, we got 99132 cells from the GB and adjacent tissue (**Figure 2A**). We then classified them into 20 clusters and annotated 10 types of cells using the Celltypist algorithm (**Figures 2B, C**), such as endothelial cells, fibroblasts, macrophages, plasma cells, T cells, B cells, monocytes, DC, ILC, and mono-mac. Cell infiltration analysis demonstrated a variety of variations between the two groups (**Figure 2D**). The representative markers of each cell type are shown in **Figure 2E**. We next assessed the glyco-score in the single-cell level leveraging the Ucell algorithm and observed a higher level of glyco-score in GB relative to the adjacent tissue, especially in macrophages and DC cells, which followed the bulk-seq results (**Figures 2F, G**).

Due to the largest proportion of macrophages in the TME of GB, we estimated the glyco-score in distinct macrophage subsets. We then filled out the macrophages and worked over again. Five subtypes of macrophages were identified, including alveolars, intermediate, erythrophagocytic, intestinal, and macrophages (**Figure 2H**). We observed that glyco-score significantly diversity among each macrophage subset (**Figure 2I**).

### 3.3 Construction and assessment of glyco-model

**Figure 3:**
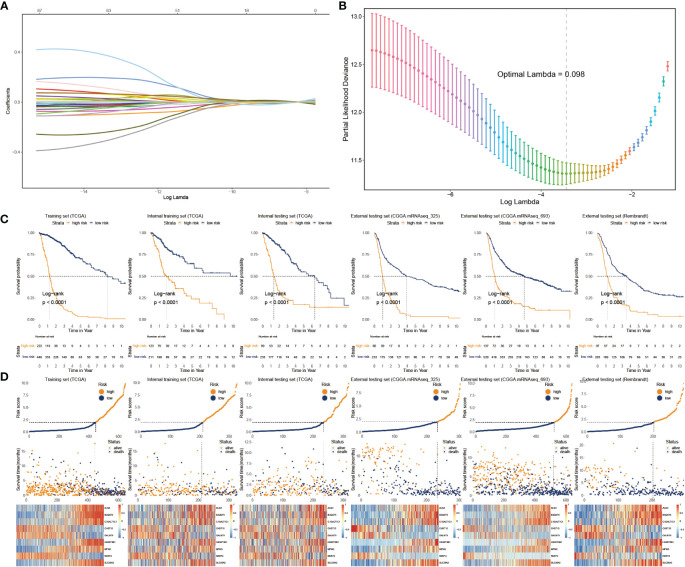
Construction and validation of glyco-model. **(A, B)** Lasso regression was used to screen out significant genes. **(C)** KM survival curve in the training sets and the testing sets. **(D)** Risk plot distribution, survival status, and relative expression of risk factors in the training and testing sets.


We applied Cox regression analysis to determine that 82 glycosylation regulators with differential expression were linked with GB prognosis (p< 0.05). Lasso regression was used to screen out significant genes. Based on the glioma cases in the TCGA dataset, 25 chosen glycosylation regulators were further analyzed to predict the risk model (**Figures 3A, B**). To strengthen the rigor of the prediction signature, we randomly divided the TCGA training set into an internal training set and an internal testing set. Then we validated the model using three independent external testing sets. After training our model, we screened nine glycosylation regulators to generate the predictive model as shown in the following formula:

$$\begin{array}{l} \text{riskscore}=\text{ALG}3\times 0.042+\text{B}3\text{GNT}5\times 0.261+\text{C}1\text{GALT}1\text{C}1\times 0.205-\text{CHST}15\times 0.411-\\ \text{GALNT}9\times 0.025+\text{HS}3\text{ST}3\text{B}1\times 0.349+\text{MFNG}\times 0.421-\text{NDST}4\times 0.117+\text{SLC}35\text{A}2\times 0.248 \end{array}$$

We categorized glioma cases into risk subtypes for survival status and time. We proved that patients from the high risk presented a considerably more proportion of deceased individuals than the low-risk subgroup. The external cohorts were categorized using the same risk signature as the training cohort. Additionally, the higher-score patients had worse outcomes and distinct gene expression profiles. KM plots demonstrated that the lower-score patients lived longer than the higher ones (**Figure 3C**). The heatmap depicts the expression characteristics of the nine chosen glycosylation regulators (**Figure 3D**).

### 3.4 Assessment of glyco-model

**Figure 4:**
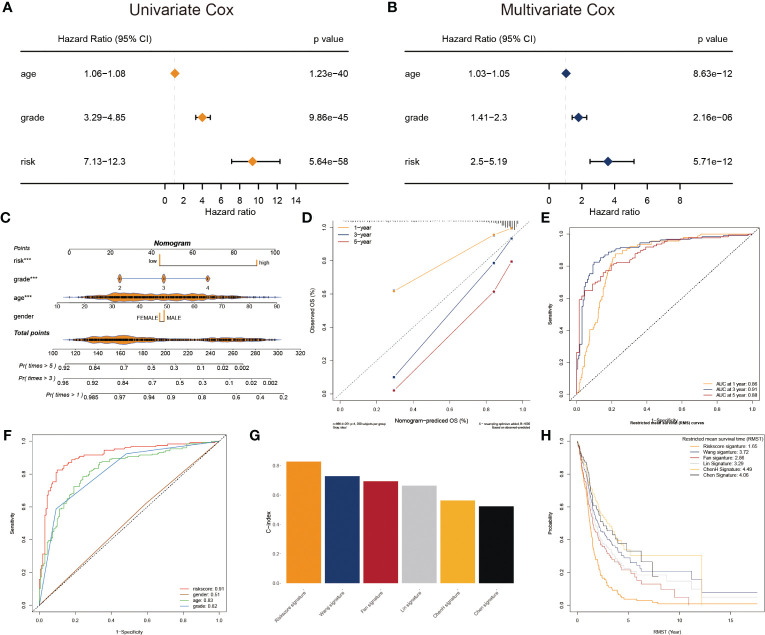
Prognostic characters of glyco-model. **(A)** The univariate Cox regression analyses the risk score and other clinical factors. **(B)** The multivariate Cox regression analyses of the risk score and other clinical factors. **(C)** Nomogram was used to predict 1-, 3-, and 5-year OS of GB. **(D)** Calibration curves were used to demonstrate the nomogram−predicted and observed OS of GB patients. **(E)** ROC curve indicating the AUC at 1-, 3-, and 5-year for the risk score. **(F)** ROC curve demonstrating the AUC of risk score and other clinical factors. **(G)** Barplot demonstrating the C-index of six risk signatures. **(H)** RMST for each of the six models.

To adapt the glyco-model for clinical application, we estimated the risk score with outcome in both univariate and multivariate analyses performed using TCGA data (p< 0.05) (**Figures 4A, B**), indicating that the risk model was reliable for GB prognosis. A nomogram was developed to estimate survival probability precisely and accurately at 1-, 3-, and 5-year, considering both the glyco-model and some standard features (**Figure 4C**). Calibration analysis verified the authenticity of nomograms in the indicated time, proving it was highly congruent with real survival time (**Figure 4D**). We further evaluated the glyco-model using the time-dependent ROC method. Survival AUCs were 0.86 (1-year), 0.91 (3-year), and 0.88 (5-year), respectively (**Figure 4E**). More so than age (AUC = 0.83), sex (AUC = 0.51), and clinical grade (AUC = 0.82), risk score (AUC = 091) AUC was a strong predictor (**Figure 4F**).

Next, we reviewed the literature and chose five current GB risk models for comparison with our novel model (28–32). Our model shows remarkable advantages over other models in C-index (**Figure 4G**) and restricted mean survival time (RMST) analysis (**Figure 4H**).

### 3.5 Significance of glyco-model to clinical features and functional deviations

**Figure 5:**
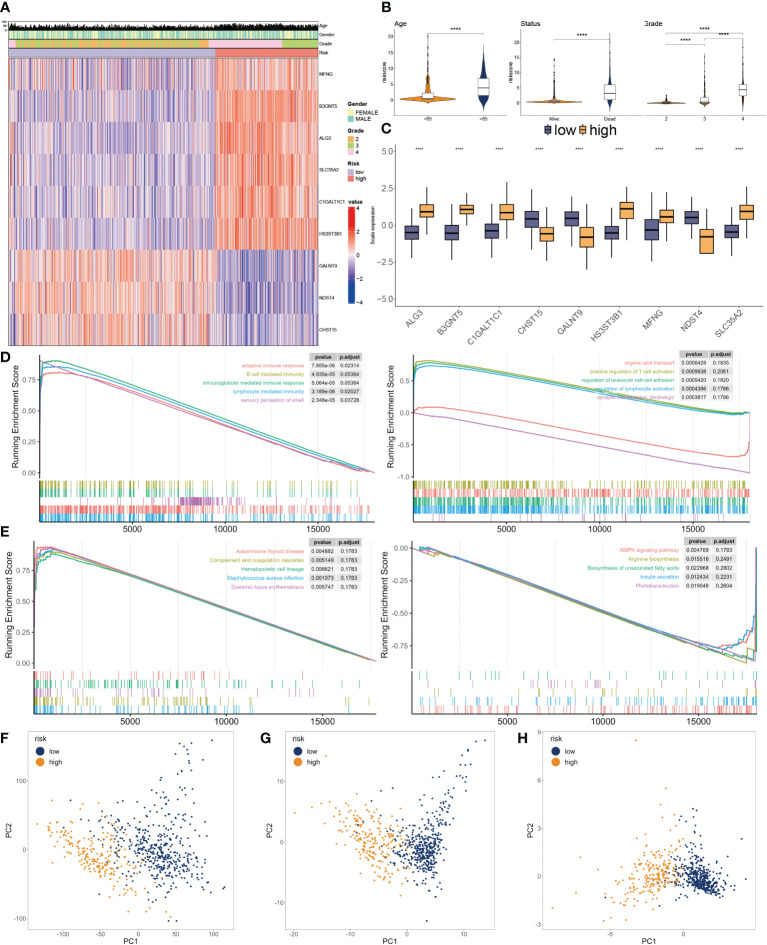
Clinicopathological characteristics of glyco-model. **(A)** Heatmap demonstrating the distribution of clinical factors (age, gender, and stage) and relative expression of nine glycosylation regulators in the two risk subgroups. **(B)** The scatter diagram of risk score and age, survival status and grade. **(C)** Relative expression of nine glycosylation regulators between the two risk subgroups. **(D)** Representative GO enrichment between the two risk subgroups. **(E)** Representative KEGG enrichment between the two risk subgroups. **(F)** PCA for all gene expression profiles. **(G)** PCA for all glycosylation regulators expression profiles. **(H)** PCA for nine glycosylation regulators expression profile. ****P < 0.001.

The heatmap plot illustrates the pattern of the nine glycosylation regulators and the clinicopathological variables (**Figure 5A**). Further analyses showed that the glyco-model positively predicts the age, OS status, and clinicopathological grade (**Figure 5B**), as indicated. We also observed positive correlations between six genes and the risk score, whereas negative correlations existed between three genes and the score (**Figure 5C**).

To investigate the dysfunctions between the two subgroups, GSEA was applied. We observed that adaptive immune response, B cell-mediated immunity, immunoglobulin-mediated immune response, lymphocyte-mediated immunity, and autoimmune thyroid disease were enriched in the higher subgroup. Moreover, positive regulation of T cell activation, regulation of leukocyte cell-cell adhesion, regulation of lymphocyte activation, and AMPK signaling pathway had inhibited activity in the lower subgroup (**Figures 5D, E**). We then conducted PCA using the entire genes (**Figure 5F**), glycosylation regulators (**Figure 5G**), and nine chosen glycosylation regulators from the model (**Figure 5H**). The outcome suggested that the expression patterns of the nine chosen glycosylation regulators effectively distinguished between the two subgroups.

### 3.6 Glyco-model correlates the tumor environment and responses to immunotherapy for GBs

**Figure 6:**
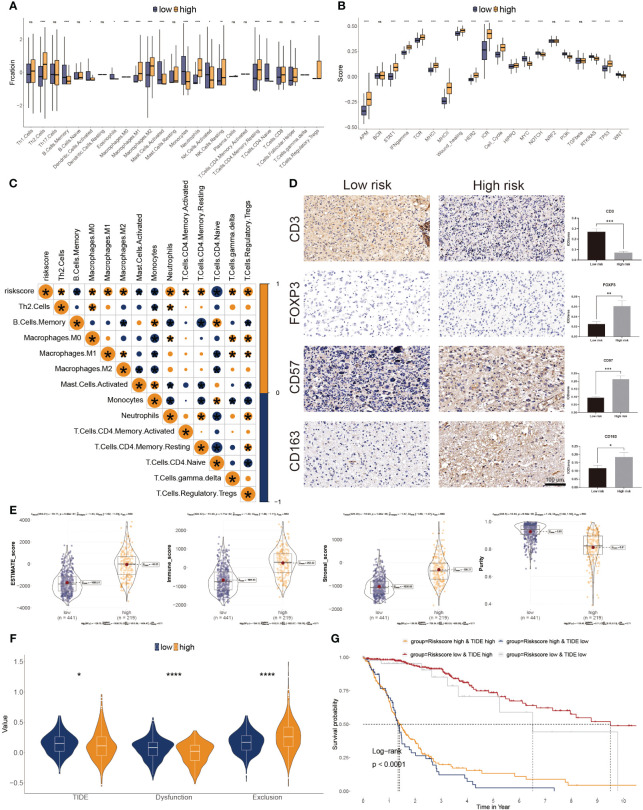
Correlation between immune infiltration and glyco-model. **(A)** Differential immune infiltration of 22 immune cell fractions between the two risk subgroups. **(B)** Pathway activities between the two risk subgroups. **(C)** The association of 22 immune cell types with the risk score. **(D)** Representative IHC images of immune cell markers between the two risk subgroups. *P< 0.05, **P< 0.01, ***P< 0.001, n.s, not significant. **(E)** Correlation of risk score with tumor microenvironment. **(F)** TIDE, T cell dysfunction, and exclusion between the two risk subgroups. **(G)** Survival analysis of patients with different combinations of risk scores and TIDE in TCGA cohort. ****P < 0.0001.

The CIBERSORT was used to calculate the 22 immune cell fractions, and ssGSEA was used to validate the score of 20 associated pathways in the two subgroups (**Figures 6A, B**). The lower-risk subtypes showed a greater abundance of the TEM cells, including activated DC cells, Eosinophils, and some types of B and T cells, but lower infiltration of M2 macrophages and Tregs. Meanwhile, 17 pathways were considerably variated between the two risk subtypes. Moreover, a significant correlation between the risk score and the proportion of immune cells was also observed (**Figure 6C**). We also confirmed the immune infiltration in glioma samples using immune cell markers (**Figure 6D**). We observed that high-risk patients had greater Tregs, tumor-associated macrophages, and NK cells but fewer T cells.

The ESTIMATE algorithm was leveraged to evaluate three scores and tumor purity. Three scores were dramatically higher in the higher risk score subtype, whereas tumor purity was lower in the lower ones, indicating immunotherapy may be less practical (**Figure 6E**). Moreover, tumor TIDE and dysfunction, but not exclusion, were significantly more prevalent in the lower risk score subtype (**Figure 6F**). On the side, individuals with higher TIDE and low-risk scores had the most favorable outcomes (**Figure 6G**).

**Figure 7:**
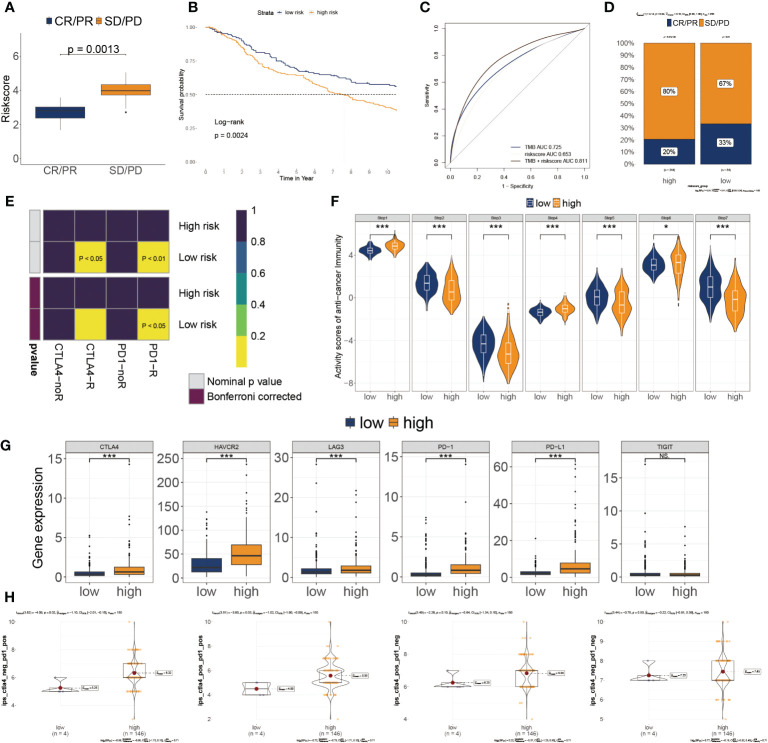
Association of risk score to the tumor microenvironment and response to immune checkpoint inhibitors. **(A)** Risk score distribution for different anti-PD-L1 clinical responses in the IMvigor210 cohort. **(B)** Survival analysis of risk score in the IMvigor210 cohort. **(C)** ROC curve of the risk score in the IMvigor210 cohort. **(D)** The relative proportion to anti-PD-L1 immunotherapy in the IMvigor210 cohort. *P< 0.05, ***P< 0.001. **(E)** Putative immunotherapy response between the two risk subgroups. **(F)** Differential immune cycle processes between the two risk subgroups. **(G)** Differential expression of six immunosuppressive molecules between the two risk subgroups. **(H)** Four subtypes of IPS values between the two risk subgroups.

Based on IMvigor210 cohorts (33), we discovered that anti-PD-L1 therapeutic response was adversely correlated with risk score (**Figure 7A**). KM plot showed that the lower risk score subtype from the IMvigor210 cohort demonstrated a better outcome for anti-PD-L1 treatment (**Figure 7B**). Moreover, the combination of TMB and risk score showed better than one of them (**Figure 7C**). The risk score was also lower in the CR/PR group relative to the SD/PD group. (**Figure 7D**). These results showed that a positive reaction to anti-PD-L1 treatment might result in a favorable outcome for the low-risk subgroup. We then employed the SubMAP algorithm to predict the anti-PD1/CTLA4 response probability of immunotherapeutic strategy between the two risk subgroups. The findings suggested that PD-1 treatment could be more effective in the lower-risk population (**Figure 7E**). However, there was no significant alteration between the two risk subgroups in anti-CTLA4 responsiveness. We further evaluated the seven steps of the immune cycle and observed substantial variations between the two types (34) (**Figure 7F**). The risk score was adversely linked with the degree of expression of immune inhibitors PD1, PD-L1, HAVCR2, LAG3, and CTLA-4 (**Figure 7G**). In addition, the low-risk subgroup was more likely to respond to monoclonal antibody therapy, such as PD-1, PD-L1, and CTLA-4 (**Figure 7H**).

### 3.7 Discovery of potential drugs for high glyco-model GBs

Genes exhibiting a robust positive correlation with the risk score could potentially hold therapeutic implications for individuals with elevated risk scores (35). Nevertheless, the majority of human proteins remain challenging to target due to their lack of distinct active sites amenable to binding with small molecule compounds, or due to their cellular localization that restricts accessibility for biological agents. As a result, the pursuit of potentially druggable therapeutic targets for GB patients grappling with dismal prognoses was initiated. In this endeavor, a compilation of target information encompassing 6,125 compounds was amassed. This was followed by a two-tiered analytical process aimed at unveiling prospective targets.

**Figure 8:**
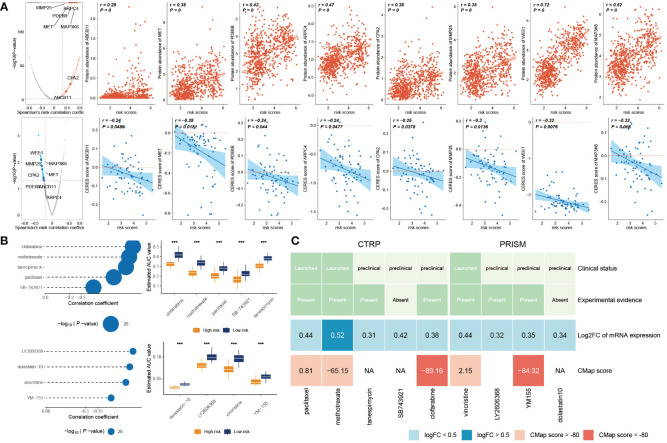
Prediction of potential drugs for high risk GB patients. **(A)** The volcano plot visually portrays the outcomes of Spearman’s correlation analysis, wherein r > 0.25 and P< 0.05 are depicted as vivid red dots. Subsequent scatter plots illustrate the correlations between the risk score and the gene abundance of identified drug targets. Analogously, Spearman’s correlation outcomes, illustrated by blue dots, signify negative associations (P< 0.05 and r< -0.2). These scatter plots depict the relationships between the risk score and the CERES score of the designated drug targets. **(B)** Compound correlation analysis and boxplot: On the left, Spearman’s correlation analysis showcases the interrelation of five compounds extracted from the CTRP dataset (top left), and the four compounds procured from the PRISM dataset (bottom left). The corresponding boxplot on the right succinctly illustrates the contrast in estimated Area Under the Curve (AUC) values across the distinct compounds within the two groups. **(C)** The diagram encapsulates a comprehensive overview, encompassing the clinical status, empirical evidence, mRNA expression levels, and CMap scores for the nine agents sourced from CTRP and the four agents derived from PRISM, respectively. ***P < 0.001.

Initially, we computed the correlation coefficient between the expression levels of druggable genes and risk score. This preliminary step yielded 607 gene targets, each marked by a correlation coefficient exceeding 0.25 (with a significance threshold of P< 0.05). Subsequently, we embarked on a parallel analysis by conducting a correlation study between the CERES score and risk score. This was predicated on glioma cancer cell lines. This supplementary analysis unveiled an additional 85 targets, distinctly associated with unfavorable prognosis (characterized by Spearman’s r< -0.2 and P< 0.05). Intriguingly, six genes—ARPC4, CPA2, MAP3K6, MET, MMP25, and WEE1—consistently emerged through both analytical approaches (**Figure 8A**).

The CTRP and PRISM datasets encompass comprehensive gene expression profiles and drug sensitivity data across a multitude of CCLs, forming an ideal foundation for constructing a predictive model for drug response. Two distinct methodologies were employed to identify candidate agents displaying elevated drug sensitivity in patients with high-risk scores. These analyses were executed employing drug response data derived from both the CTRP and PRISM datasets.

In the initial step, a comparative analysis of differential drug response was undertaken between the high-risk score (top decile) and low-risk score (bottom decile) groups. The objective was to identify compounds demonstrating lower estimated AUC values within the high-risk score group (with a log2 fold change > 0.2). Subsequently, a Spearman correlation analysis was conducted between AUC values and risk scores. This facilitated the selection of compounds showcasing a negative correlation coefficient (Spearman’s r< -0.30 for CTRP or -0.35 for PRISM). Through this approach, a total of six compounds derived from CTRP (including clofarabine, SB743921, tanespimycin, methotrexate, and paclitaxel) and four compounds derived from PRISM (including dolastatin10, YM155, LY2606368, and vincristine) emerged. Importantly, all these compounds exhibited reduced estimated AUC values within the high-risk score group and a negative correlation with risk score as demonstrated in **Figure 8B**.

Despite the observation that the identified 10 candidate compounds displayed heightened drug sensitivity in high-risk score patients, it is crucial to acknowledge that these analyses in isolation do not substantiate the therapeutic efficacy of these compounds in the context of GB. Consequently, an array of multifaceted analyses was subsequently undertaken to delve into the therapeutic potential of these compounds within GB. Firstly, the CMap analysis was employed to identify compounds whose gene expression patterns ran counter to those specific to GB (characterized by increased gene expression in tumor tissues yet attenuated by treatment with particular compounds). Notably, two compounds—clofarabine, and YM-155—secured CMap scores below -80. This inference suggests the potential therapeutic impact of these compounds in GB. Secondly, a thorough literature review was undertaken on PubMed to ascertain experimental and clinical evidence about the efficacy of candidate compounds in GB treatment. The cumulative outcomes of these analyses were depicted in **Figure 8C**.

Overall, on a broad scale, clofarabine and YM-155 exhibited robust *in vitro* and silico evidence, positioning them as the most promising therapeutic contenders for GB patients with elevated risk scores.

## 4 Discussion

GB is hard to combat, and its prognosis varies based on the molecular subtypes. To evaluate the prognosis of GB, there is a critical need for unique and effective techniques. This work effectively constructed a risk signature based on nine glycosylation regulators screened: ALG3, B3GNT5, C1GALT1C1, CHST15, GALNT9, HS3ST3B1, MFNG, NDST4, and SLC35A2. We also showed that patients from high-risk subgroups based on target glycosyltransferases are strongly related to a shorter OS, a poorer immunological impact, and greater chemosensitivity than the lower subgroup. Glycosyltransferases are a vast group of enzymes that regulate glycosylation and promote tumor development and metastasis. In this work, each of the nine glycosyltransferases in our model has unique properties and roles.

Glycosylation-related regulators are highly effective diagnostic tools for early cancer diagnosis, grade identification, and therapy methods. Mohamed et al., for instance, developed a glyco-model by evaluating glycosylation regulators’ expression patterns that may be used to distinguish pancreatic cancer subtypes (36). Additionally, the expression of glycosylation regulators may aid the detection of CTCs in cancer patients’ blood samples utilizing PCR (11). Although various glycosylation regulators demonstrated adequate and satisfactory consequences in the risk model, no notable features may suggest the presence of CTCs in the blood (37). Therefore, their clinical applicability requires further development. However, the predictive utility of glycosylation regulators has been studied before (38, 39), usually just by looking at a single gene rather than a set of genes together, as we did in the GB.

Moreover, there has been little bioinformatics-based research on the prognostics of GB connected with the glycosyltransferase gene. The glycosylation-based model was able to differentiate between the risk subgroups in our elaborate work. The lower risk subgroup was significantly correlated with longer OS compared to the higher ones, indicating that our model may accurately predict the outcomes of GB patients.

We then identified the clinical characteristics and prediction of GB. The importance of glycosylation in modulating immune-related function and anticancer immunity is growing. The essential glycosyltransferase, such as selectins, singles, and galectins, are crucial regulators of the immune response in tumor spread (40). Numerous immune response-related signals were abundant in the higher-risk score patients. The immunological and stromal scores were significantly higher in the high-risk patients, although tumor purity was considerably elevated in the lower-score patients. Wang et al. found that the glycosyltransferase gene ADRB1 is a significant immunotherapy biomarker among gene mutations (41).

Additionally, we found that tumor glycosylation was significantly correlated with the expression of immunological checkpoints. Current research indicated the higher expression of PD1/PD-L1, the more sensitive to immunosuppressive therapy. Similar to our results, the group with a low score was more responsive to anti-PD-1/PDL1 treatment in the IMvigor210 cohort. We found that the low-risk subset of GB patients may respond better to PD-1 therapy. Nonetheless, the two risk subtypes failed to respond to CTLA4 immunotherapy. The high-score group also showed greater drug sensitivity than the lower ones. Hence, we found significant differences in the prediction of chemotherapy response. We expect the risk score to differentiate between the two risk groupings and provide more accurate predictions about the efficacy of anti-PD1 or anti-PD-L1 immunotherapy treatments. One hypothesis about the effectiveness of ICI for GB is that therapy is more likely to assist those with a low-risk score.

However, specific issues still need to be resolved in the present research. Firstly, this is retrospective research primarily created using bioinformatics analysis of TCGA information and IMvigor210. Validation of the clinical predictive validity of this well-established glyco-signature is still absent. In the future, adequate external verifications should be conducted. Second, we solely validated the immune cell infiltration of the GB samples using the IHC test. These validated results were insufficient to account for all anticipated consequences. These glycosylation regulators’ multiple functions and essential mechanisms in GB oncogenesis, development, and prognosis remain deciphered. To corroborate the risk score model’s originality, future prospective studies evaluating a large and multicenter population may be advantageous.

## 5 Conclusion

To sum up, we mined the TCGA database for nine glycosylation regulators and used them to build a functional glyco-model. The immunosuppression and prognosis of the high-risk category were shown to be worse. Immune cell invasion, the tumor-immune cycle, the ICI response, and chemosensitivity in GB are all interconnected with this glyco-model. Investigating glycosylation regulators levels in GB patients might improve our understanding of TME and aim for the design of more efficient therapy protocols. The prognosis for treating GB can be significantly enhanced by integrating our glyco-model with the standard gold techniques.

## Data availability statement

The original contributions presented in the study are included in the article/supplementary material. Further inquiries can be directed to the corresponding author.

## Ethics statement

The studies involving humans were approved by Ethics Committee of China-Japan Union Hospital of Jilin University. The studies were conducted in accordance with the local legislation and institutional requirements. The participants provided their written informed consent to participate in this study.

## Author contributions

XJ: Writing – review & editing, Writing – original draft, Visualization, Validation, Resources, Methodology, Investigation, Funding acquisition, Formal analysis, Data curation, Conceptualization. ZC: Writing – original draft, Visualization, Resources, Project administration, Methodology, Investigation, Formal analysis. HZ: Writing – review & editing, Writing – original draft, Validation, Resources, Formal analysis, Data curation, Conceptualization.
